# Pseudohernia Following Herpes Zoster Presenting With Transient Abdominal Wall Bulging: A Case Report

**DOI:** 10.7759/cureus.91076

**Published:** 2025-08-26

**Authors:** Nagatani Kei, Tsunoda Takahiko

**Affiliations:** 1 Dermatology, Yamagata City Hospital Saiseikan, Yamagata, JPN

**Keywords:** herpes zoster incidence, neurogenic paralysis, pseudohernia, segmental zoster paresis, herpes zoster

## Abstract

Herpes zoster (HZ), caused by reactivation of latent varicella-zoster virus within the sensory ganglia, typically presents with painful vesicular eruptions. While motor nerve involvement is uncommon, it can occasionally lead to abdominal wall pseudohernia. Pseudohernia results from denervation and paralysis of abdominal muscles in the absence of a fascial defect, and is easily mistaken for a true hernia. We report the case of a 78-year-old man who developed a left-sided abdominal wall bulge shortly after the onset of HZ rash in the T10-T12 dermatomes. The bulge became prominent when standing but diminished in the supine position. CT revealed asymmetric thinning of the internal oblique and transversus abdominis muscles at the T11 level, with preservation of fascial continuity and no protrusion of intra-abdominal contents. These findings, combined with the close anatomical correspondence between the skin rash and the innervation of affected muscles, supported the diagnosis of HZ-related pseudohernia. Differential diagnoses such as diabetic truncal neuropathy, postsurgical changes, scarring myopathy, and localized fat atrophy were considered but excluded based on clinical history and imaging. The patient was managed conservatively with antiviral therapy and neuropathic pain control, leading to complete resolution of the bulge within three months. This case emphasizes that when abdominal wall bulging occurs near recent HZ skin lesions, pseudohernia should be strongly suspected. Recognition of this condition is essential to avoid misdiagnosis and unnecessary surgical procedures. Diagnosis can be supported by both clinical findings, including reduction of the bulge in the supine position, and imaging evidence of muscle thinning without a hernial defect. Prognosis is generally favorable, as most patients recover with conservative management alone.

## Introduction

Herpes zoster (HZ) results from the reactivation of varicella-zoster virus (VZV), the pathogen responsible for chickenpox in childhood. After the initial infection, the virus remains latent in the sensory ganglia, clusters of nerve cell bodies that transmit sensation from the skin, for decades [1]. Reactivation occurs when host immunity declines with age, stress, or immunosuppression [1]. The annual incidence of HZ is estimated at 4-4.5 cases per 1,000 person-years, and approximately 20-30% of individuals will develop HZ during their lifetime [2].

HZ typically involves sensory nerves, producing localized pain and vesicular eruptions along dermatomes. In rare cases, the virus spreads from the dorsal root ganglia to the anterior horn cells and along motor fibers, leading to the denervation of targeted muscles and neurogenic paralysis [1,3]. Involvement of thoracic segments T6-T12 can weaken the rectus abdominis, internal oblique, and transversus abdominis muscles, allowing intra-abdominal pressure to produce a visible bulge despite an intact fascial wall.

A true hernia is defined by the presence of a hernial defect, a measurable disruption in the abdominal wall fascia through which intra-abdominal contents protrude, often contained within a hernia sac. In contrast, a pseudohernia presents as a similar bulge but results solely from muscle weakness or paralysis due to motor nerve injury, without any fascial defect [3,4]. Clinically, both may present as localized protrusions that become more pronounced when standing or coughing. However, in pseudohernia, the bulge typically disappears or markedly reduces in the supine position, and CT or ultrasound demonstrates thinning of the abdominal muscles with preserved fascial continuity [4-6]. Recognizing these differences is essential, as correct diagnosis can prevent unnecessary surgical intervention.

Motor complications of HZ occur in only 0.5-5% of cases, with abdominal wall pseudohernia being even rarer, affecting approximately 0.17% of patients [2,3]. This rarity is likely due to the small proportion of motor fibers in mixed spinal nerves, the virus’s preference for sensory neurons, and overlapping segmental innervation of the abdominal wall, which can mask mild paresis [1,3,4].

The standard treatment for HZ includes early initiation of antiviral agents such as acyclovir, valacyclovir, or amenamevir, ideally within 72 hours of rash onset, combined with adequate pain control to reduce acute symptoms and the risk of postherpetic neuralgia. In cases with motor involvement, management remains conservative, focusing on viral suppression, analgesia, and observation for spontaneous recovery.

Here, we report the case of a 78-year-old man who developed transient left-sided abdominal wall bulging secondary to segmental motor paresis following HZ, and we review the epidemiology, pathophysiology, diagnostic approach, and clinical relevance of HZ-related pseudohernia.

## Case presentation

**Figure 1:**
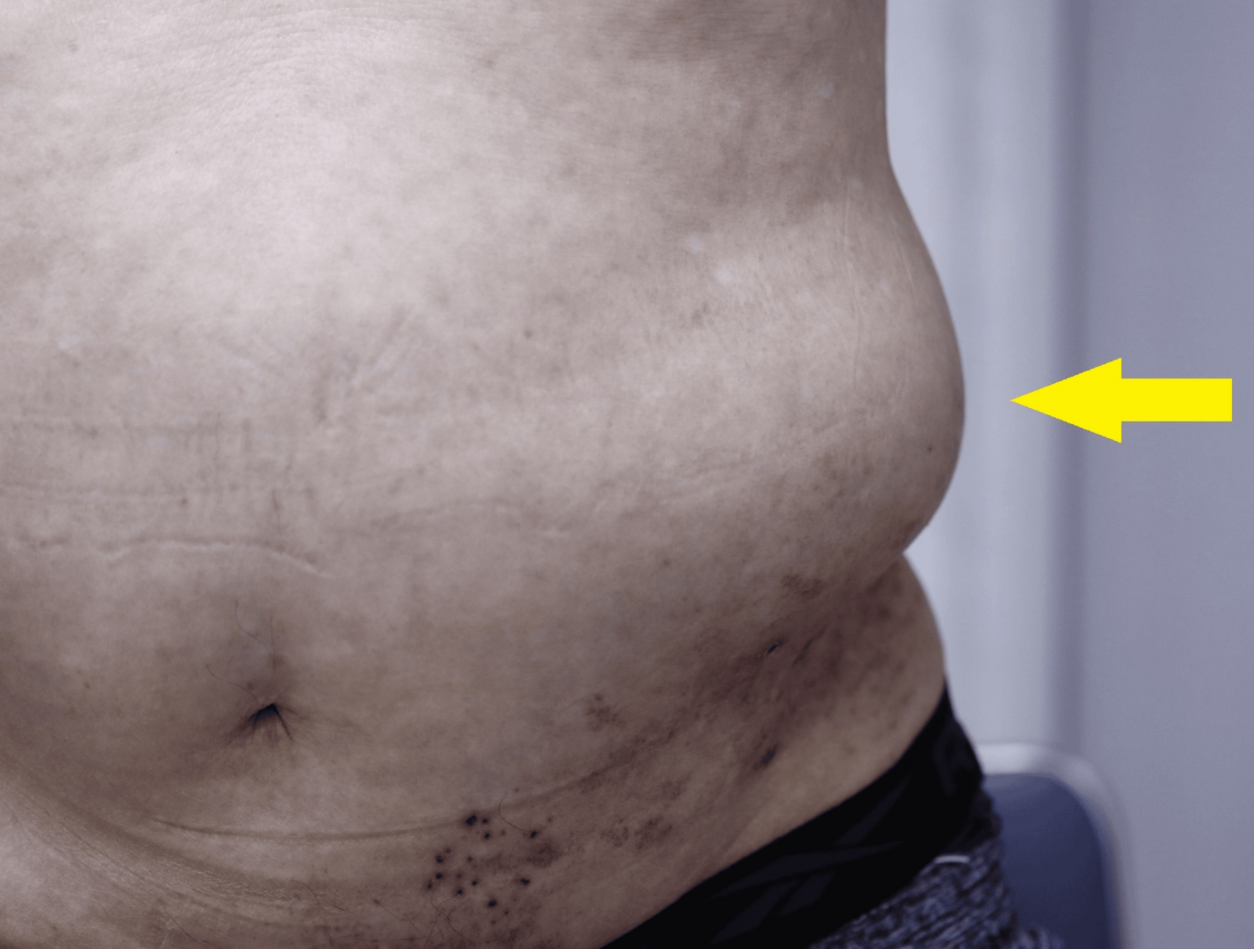
Abdominal wall bulging mimicking hernia following herpes zoster. Photograph of the patient’s left abdomen taken in the standing position. A soft, localized bulge is visible (arrow) just above the site of prior herpes zoster eruptions. The bulge disappeared in the supine position, consistent with pseudohernia secondary to segmental zoster paresis.

A 78-year-old man developed HZ in 20XX and subsequently presented with a new bulge on the left flank. His past medical history included an acute myocardial infarction treated with stent placement in 20XX-20 and retinal detachment surgery in 20XX-18. He had quit smoking more than 20 years earlier and had no history of diabetes, abdominal surgery, trauma, or neuromuscular disease. He occasionally consumed alcohol, and there was no family history of similar conditions. On August 29, 20XX, he developed pain and vesicular eruptions extending from the left flank to the lower thorax. He was diagnosed with HZ at a local clinic, and amenamevir was initiated within 48 hours of rash onset for a seven-day course. Pain was managed with neurotropin, mecobalamin, and pregabalin. Approximately 10 days after onset, a gradually progressive bulge appeared at the same site. On September 11, 20XX, he first visited our hospital, where physical examination revealed crusting and post-inflammatory hyperpigmentation involving the left T10-T12 dermatomes. He reported tingling neuralgic pain in the same dermatomes, which was mild and controlled with oral medications, and no distinct sensory deficits were apparent. A soft, compressible, infant head-sized bulge was observed on the left flank (Figure 1).

**Figure 2:**
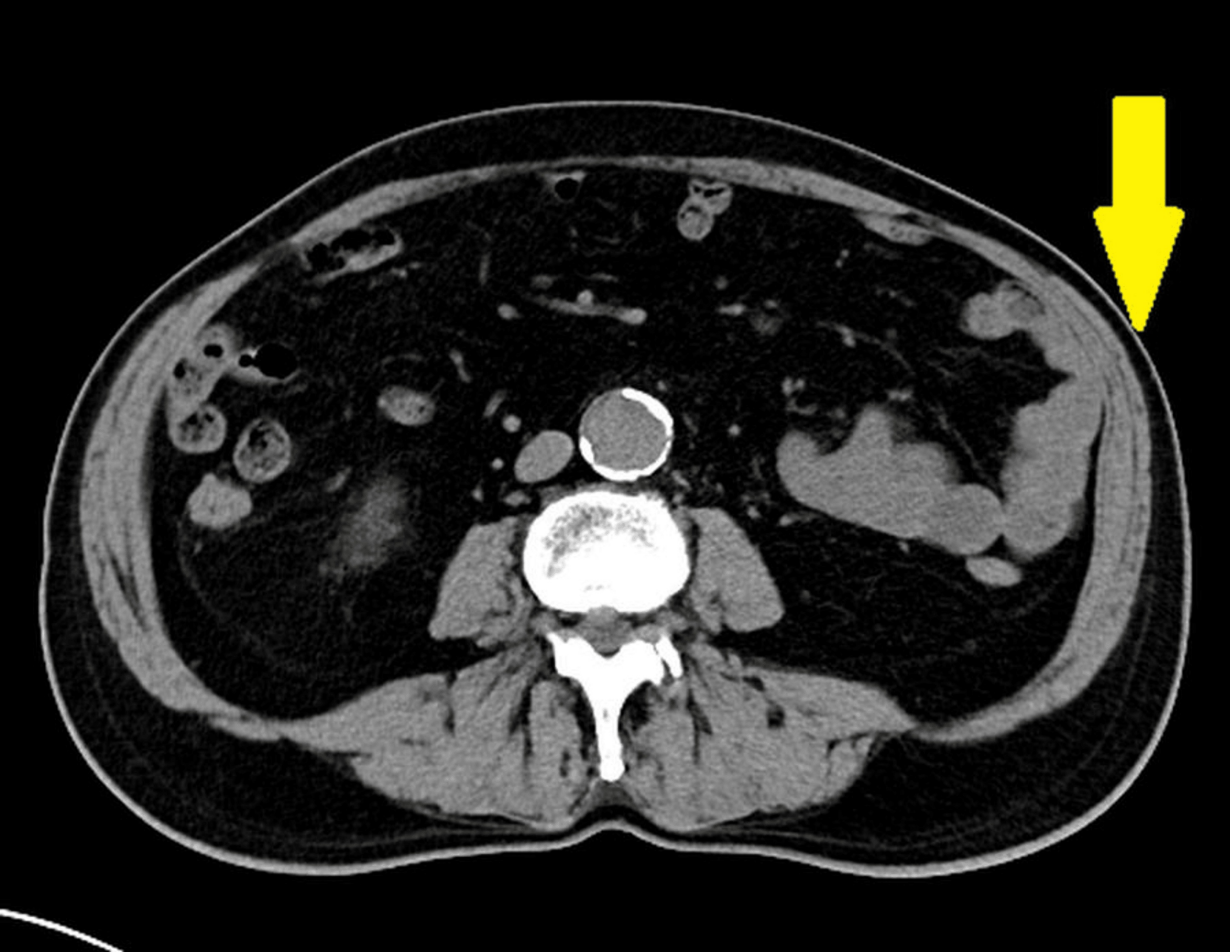
Abdominal muscle thinning on the left side following herpes zoster. CT image showing asymmetric thinning of the left abdominal wall muscles (arrow), consistent with segmental motor paresis following herpes zoster infection. No evidence of true herniation is observed.

It was most prominent in the standing position and markedly reduced in the supine position. The contralateral abdominal wall was normal, and no fascial defect was palpable. Abdominal CT performed in both supine and prone positions demonstrated asymmetric thinning of the left internal oblique and transversus abdominis muscles at the level corresponding to T11, with preservation of fascial continuity and no protrusion of intra-abdominal contents (Figure 2).

**Figure 3:**
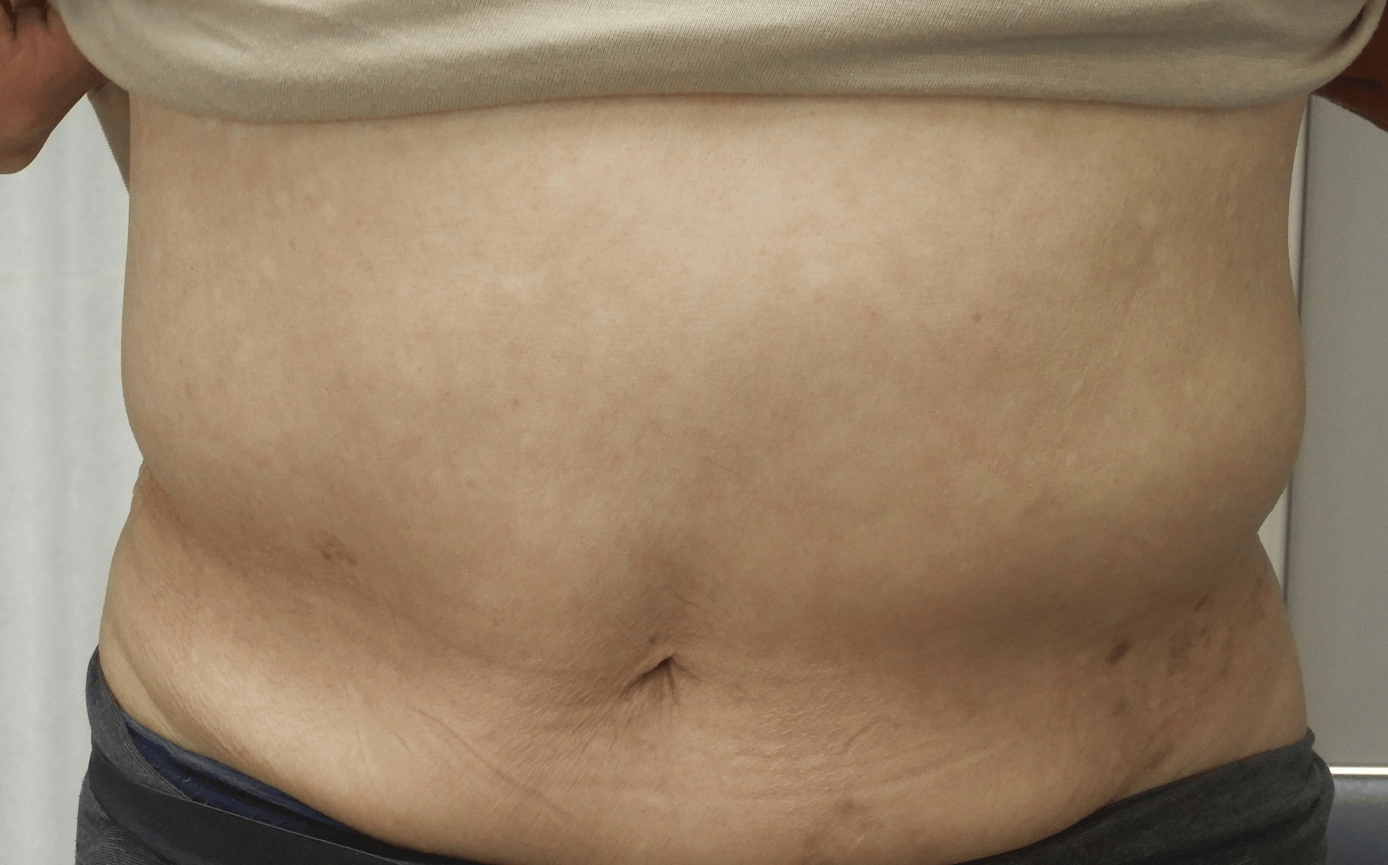
Clinical image after symptom improvement. Clinical photograph obtained three months after onset, showing complete resolution of the left-sided abdominal wall bulge. The left flank bulge has nearly disappeared, and the abdominal wall has improved to the same level as the unaffected side. The residual post-herpetic pigmentation on the left flank has also markedly improved compared with the initial presentation.

The right abdominal wall muscles appeared normal, and no intra-abdominal mass or fluid collection was detected. These findings ruled out a true hernia, as no fascial defect or bowel protrusion was present, and also excluded pneumatosis intestinalis. Given the temporal association with HZ onset, the reduction of the bulge in the supine position, and imaging findings of muscle thinning without fascial defect, a diagnosis of abdominal wall pseudohernia due to segmental motor paresis secondary to HZ was made. There was no history of diabetes, abdominal surgery, or trauma, which also excluded the possibility of diabetic truncal neuropathy and scar-related myopathy. Antiviral therapy and medications for neuropathic pain were continued, and the patient was followed conservatively. By December 2, 20XX, the bulge had completely resolved, with no residual motor or sensory deficits (Figure 3).

## Discussion

Segmental paralysis associated with HZ is a rare complication, with an estimated incidence of 0.5-5% [1]. Among these cases, pseudohernias resulting from abdominal muscle paralysis are even more infrequently reported [2-4]. Similar to previous reports, our patient was an elderly male who presented with localized abdominal bulging, demonstrated no evidence of herniation on imaging, and improved with conservative management [4,7,8].

Pathophysiologically, VZV spreads from the dorsal root ganglia to the anterior horn cells and motor nerves, leading to denervation of the abdominal muscles innervated by the T6-T12 segments [1,3]. In some reports, MRI has demonstrated gadolinium contrast enhancement of the affected anterior nerve roots, providing radiologic evidence that supports direct viral involvement of motor pathways [9]. These abdominal wall muscles are supplied by the intercostal nerves, which are mixed nerves providing both motor innervation to the rectus abdominis, external and internal oblique, and transversus abdominis muscles, as well as sensory innervation to the corresponding dermatomes [1]. Because of overlapping innervation among adjacent segments, mild motor involvement may not be clinically apparent, which explains the rarity of symptomatic abdominal wall paralysis despite frequent zoster reactivation [1,2]. In our case, abdominal CT revealed asymmetric thinning of the left internal oblique and transversus abdominis muscles at the T11 level, while the rectus abdominis was relatively preserved. This pattern corresponds well to the segmental innervation of the abdominal wall: although all these muscles are supplied by the intercostal nerves, the internal oblique and transversus abdominis also receive contributions from the iliohypogastric and ilioinguinal nerves, allowing for partial sparing and overlap. Such anatomical considerations further support that localized denervation by VZV can manifest as pseudohernia in the absence of fascial defects [2-4].

On CT, muscle thinning or mild bulging without a hernia sac is typically observed, as was confirmed in our case [8,9]. In particular, the absence of intra-abdominal contents protruding through the fascia allowed us to clearly rule out a true hernia and intestinal pneumatosis. Although these are based on case reports, previous studies have also described similar CT findings, including thinning of the abdominal muscles, absence of structural defects such as true hernias or masses, and the utility of CT in differentiating pseudohernia from other conditions, which support the usefulness of CT in the diagnosis of HZ-induced pseudohernia. In addition, ultrasonography has been reported to provide a diagnostic advantage over CT in detecting subtle thinning of the abdominal wall muscles. Importantly, ultrasonography should be performed bilaterally, as assessing only the affected side makes it impossible to appreciate differences in muscle thickness. With its high spatial resolution, low invasiveness, and repeatability, ultrasonography represents a valuable adjunctive tool in the evaluation of zoster-related pseudohernia [10]. Although electromyography (EMG) was not performed in the present case, its use as a diagnostic adjunct has been proposed, with studies showing that EMG can detect motor paresis more frequently than clinical examination alone (36% vs. 19%) [11]. This suggests that EMG may be particularly useful in identifying subtle motor deficits or in cases where the diagnosis is unclear. Additionally, MRI may reveal findings such as contrast enhancement of the affected nerve roots or denervation changes in the abdominal muscles, which can be helpful in cases where the diagnosis is challenging [9,12].

As for the differential diagnosis, diabetic truncal radiculopathy, localized fat atrophy, scarring myopathy, and postsurgical changes were considered. However, the absence of a history of diabetes, abdominal surgery, or trauma allowed us to exclude these possibilities. Importantly, in our patient, the development of abdominal wall bulging closely followed the appearance of HZ rash and gradually resolved in parallel with the improvement of skin lesions and pain. This chronological association strongly supports the diagnosis of zoster-related pseudohernia rather than alternative etiologies.

Conservative management, including antiviral therapy and pain control, is generally effective, with approximately 70% of cases showing complete recovery and an additional 17% demonstrating improvement within three months [2]. This favorable outcome is not limited to truncal segmental zoster paresis; similarly high recovery rates have also been reported in cases involving the limbs and trunk [1]. In our patient, the favorable prognosis may be attributed to the absence of diabetes or immunosuppressive conditions, the limited extent of cutaneous lesions, and the initiation of appropriate conservative treatment in the early stage. However, our patient had no prior vaccination for HZ, which highlights the importance of vaccination in preventing recurrence.

## Conclusions

Pseudohernia is a rare but important differential diagnosis in patients presenting with abdominal wall bulging following HZ. Recognizing this condition is essential to prevent misdiagnosis and avoid unnecessary surgical intervention, especially when imaging does not show a clear hernia. When abdominal wall bulging occurs in close proximity to HZ skin lesions, pseudohernia due to HZ should be strongly considered. Diagnosis can be supported not only by clinical findings, such as the disappearance of the bulge in the supine position, but also by correlating imaging findings of muscle thinning with the motor innervation of the dermatome affected by the rash. Conservative treatment with antiviral agents and pain control is usually sufficient, and the prognosis is generally favorable.
